# Towards Sustainable Antibiotic Use in Aquaculture and Antimicrobial Resistance: Participatory Experts’ Overview and Recommendations

**DOI:** 10.3390/antibiotics13090887

**Published:** 2024-09-14

**Authors:** Daniela R. Farías, Rolando Ibarra, Rodrigo A. Estévez, Michael F. Tlusty, Oskar Nyberg, Max Troell, Ruben Avendaño-Herrera, Wendy Norden

**Affiliations:** 1 Monterey Bay Aquarium Global Oceans Conservation Program, 886 Cannery Row, Monterey, CA 93940, USA; ribarra@mbayaq.org (R.I.); michael.tlusty@umb.edu (M.F.T.); wnorden@mbayaq.org (W.N.); 2 Centro de Investigación e Innovación para el Cambio Climático, Facultad de Ciencias, Universidad Santo Tomás, Santiago 8370003, Chile; restevezw@santotomas.cl; 3 Instituto Milenio en Socio-Ecología Costera, Santiago 8320000, Chile; 4 School for the Environment, University of Massachusetts Boston, Boston, MA 02125, USA; 5 Stockholm Resilience Centre, Stockholm University, 106 91 Stockholm, Sweden; oskar.nyberg@su.se (O.N.); max@beijer.kva.se (M.T.); 6 Beijer Institute of Ecological Economics, Royal Swedish Academy of Sciences, 104 05 Stockholm, Sweden; 7 Laboratorio de Patología de Organismos Acuáticos y Biotecnología Acuícola, Facultad de Ciencias de la Vida, Universidad Andrés Bello, Viña del Mar 8370035, Chile; ravendano@unab.cl; 8 Interdisciplinary Center for Aquaculture Research (INCAR), Viña del Mar 2531015, Chile; 9 Centro de Investigación Marina Quintay (CIMARQ), Universidad Andrés Bello, Quintay 2340000, Chile

**Keywords:** antimicrobials, environmental impacts, sustainability

## Abstract

Notably, 56 worldwide experts gathered for the Antimicrobial Assessment on Global Aquaculture Production (AGAP) series of workshops to (1) evaluate the current state of knowledge on antimicrobial use and identify existing gaps; (2) formulate strategies to identify ecologically relevant impact indicators and establish thresholds for assessment; (3) identify pivotal socioeconomic factors and effective governance mechanisms essential for implementing monitoring practices in aquaculture and extending them across sectors and countries for aquaculture sustainability; (4) develop pathways to enhance our comprehension between antibiotic use in aquaculture and antimicrobial resistance; and (5) explore potential antibiotic monitoring tools that can be universally adapted and implemented across region and sectors. The main outcomes were a roadmap for establishing investigation priorities on the relevant topics regarding antibiotic use in aquaculture, socioeconomic drivers for using antibiotics and behaviors that need more robust and transparent regulatory frameworks to guide farmers, training on antimicrobial use, and access to veterinarians and extension services agents for education. Overall, the workshop evidenced the power of collaboration in addressing complex global challenges to achieve sustainable aquaculture. Despite diligent efforts, some constraints may have inadvertently narrowed the possibility of having more experts and left some pertinent topics unaddressed, but they are needed in the discussion.

## 1. Introduction

***Preamble*:** Due to the lack of data and understanding of the impact of antibiotics in aquaculture at different levels, we called an expert group to fill these gaps and provide recommendations to address this challenge as concerted efforts are required by diverse actors to formulate appropriate solutions. In this context, this study presents the outcomes of a series of workshops designed to foster global collaboration and knowledge sharing on the relevant topic of antibiotic use in aquaculture. The virtual event covered various aspects of antibiotic use in aquaculture, providing valuable networking opportunities for institutions and experts worldwide.

The United Nations (UN) 2030 Agenda for Sustainable Development recognizes aquaculture as a vital contributor to both livelihoods and food security, with its production anticipated to surge in the upcoming years [1,2]. Animal health and welfare are the foundation for the sustainability of aquaculture operations [3]. Climate change and inadequate management practices heightened the vulnerability of farmed species to pathogens [4], something that worries aquaculture producers worldwide [5,6]. Disease control measures include using antimicrobials (AMs), often without proper knowledge or consideration for potential hazards to human health and the ecosystem. AMs encompass a broad spectrum of agents, including antiseptics, antifungals, antibiotics (ABs), and other substances employed to remove pest species microorganisms. ABs have recently become the primary choice for controlling bacterial diseases in aquaculture [7,8].

Many aquaculture operations (i.e., finfish and crustacean aquaculture at all scales) may involve the overuse and misuse of AMs (e.g., growth promotion instead of disease control) [9,10,11,12,13,14,15]. It is estimated that as much as 80% of ABs administered in fish are wasted in the water, which spread rapidly through water systems [16,17,18]. This is not just in uneaten feed, but overuse increases the spread of the un-metabolized chemicals into the aquatic environment via animal waste [19,20,21]. This uncontrolled release leads to the widespread dissemination of AMs in aquatic environments where AMs are developed by non-target organisms, contributing to antimicrobial resistance (AMR) [22,23]. Undoubtedly, a better understanding of the role that aquaculture plays in AMR, as well as the current antimicrobial use (AMU) and the role that aquaculture production plays in developing AMR, is necessary given that AMR represents a critical global health concern [24]. The World Health Organization (WHO) estimated that, by 2050, economic losses for treating human diseases concerning AMR will be USD 1 trillion [25,26]. Addressing these challenges demands a multifaceted approach, encompassing improved stewardship of AMU in aquaculture, heightened awareness among stakeholders, and robust regulatory frameworks to safeguard both human health and environmental integrity.

According to Thornber et al. [13], AMs are frequently employed in some countries for prophylactic purposes to prevent infections and treat existing infections. The global shrimp aquaculture industry has commonly used AMs, which are often used in the absence of preventive tools such as vaccines or appropriate health management [27]. ABs are used in hatcheries for treating common infections such as hepatopancreatic necrosis disease (AHPND), an infection linked to the bacteria with *Vibrio parahaemolyticus* [28]. In finfish aquaculture, ABs are frequently utilized to treat common bacterial infections; in the case of Tilapia (Cichlids, Perciforme), they were used in farms to treat *Edwarsiella* sp., and in the case of salmonids, they were used to control *Piscirickettsia salmonis* and *Renibacterium salmoninarum*, which caused significant losses [17,29,30]. These pathogens are common and affect animal health and welfare, often resulting in mortality and substantial economic losses. As such, AMs have emerged as the primary and most effective method for managing bacterial disease, but this leads to many occasions of overuse.

AMs entering the environment end up in sediment and water, posing risks to non-target organisms and promoting the development of AMR and possibly accumulating in the food chain [22,31,32], but such interactions in the aquatic environment are still poorly understood [29]. For instance, in freshwater aquatic ecosystems, cyanobacteria and bacteria are two of the particularly vulnerable non-target organisms likely to be impacted by AM exposure [33]. Furthermore, AMs may negatively affect the ecosystem and its functions [34,35] by disrupting fundamental biogeochemical cycles. AMs can be excreted by animals in their active form and therefore may persist in the environment for extended periods [20,35,36].

Regardless, AM use in farmed food challenges people’s behaviors and practices for maintaining health versus company profit [37]. As an example, a study conducted in China by Shao, Wang, Yuan, and Xie [15] highlighted the weak regulations on acquiring AMs, while Ali et al. [38] demonstrated that, in Bangladesh’s shrimp farms, several chemicals are handled without protection and contribute to human health risk. Similarly, in Vietnamese finfish farms, ABs are bought in cartridges, used by crushing the pills, and operated by bare hands; however, AB application practices differ by farms and farmers [39]. The implementation of health management practices or biosecurity is not always according to the best practices recommended by the World Organization for Animal Health (WOAH).

The WOAH [40] has classified ABs according to an “AWaRe” rank, based on “Access” (ABs with a wide range of encountered susceptible pathogens with low resistance, normal AB use), “Watch” (ABs with high-resistance potential, which is a concern), and “Reserve” (ABs that should be reserved for treatment due to multidrug resistance, as well as ABs that should not be otherwise used). From the “watch list” of emerging contaminants that impact aquatic environments, amoxicillin and ciprofloxacin are two being used in aquaculture in places such as Bangladesh [8,40,41]. Other ABs, from the tetracycline group, are the most frequently used in animals and humans because of their effectiveness against a broad spectrum of bacterial pathogens, with oxytetracycline being regularly used in aquaculture [42,43,44,45,46]. However, in general, the occurrence and effects of ABs in the environment are still uncertain [8,46].

The persistence of AMs in the environment differs depending on factors such as the drug’s pharmacokinetic profile or physicochemical properties (i.e., adsorption capacity and binding, photostability, leachability, and degradation rate) [20,47]; as an example, certain ABs such as florfenicol, which is approved for use in aquaculture [48], may exhibit poor bioavailability after oral administration (e.g., LC-50 48 h observed in Tilapia), making it easier to enter the environment [49,50]. Given the impact of ABs on the environment and the subsequent development of resistance, it is fundamental to understand AB degradation.

The Food and Agriculture Organization (FAO) has highlighted several challenges such as poor or missing monitoring practices, the lack of harmonization of testing and sampling methods, and the low transparency of data, which hinder comparisons across regions [7,51]. Indeed, the absence of global standards for evaluating the ecological effect of AMU in aquaculture represents a significant challenge. To address these challenges, the FAO [52] has published a set of guidelines for the monitoring and surveillance of AMR in agriculture for some Asian countries that have been gradually implemented. These guidelines tailored sampling methods for AMR monitoring and surveillance, target population and specific pathogens (determined by each country), and surveillance approach (focusing on diseased/infected aquatic species). More recently, the UN incorporated the Program of United Nations for the Environment (PNUMA) into the existing tripartite alliances FAO-WOAH-WHO, now quadripartite FAO-WOAH-WHO-UNEP, to accelerate the management strategy for progress in this area, thus improving coordination among human–animal ecosystems [52].

From a methodological point of view, different analytical methods for assessing AMR have been developed in animal farms; however, they have been context-specific, with restricted applicability for production schemes across regions or considering species that have not been applied in aquaculture. Therefore, there is a critical need to expand the understanding of AMU to establish standards for sampling methods and tools [53]. While methodologies exist for assessing the impacts of AMU on human health, equivalent sampling protocols for assessing ecological impacts remain lacking [54], underscoring the importance of further research and development in this area.

On account of the concerns related to antibiotic use (ABU) and AMR, adopting the One Health approach, an integrated approach considering the health of people, animals, and ecosystems, is crucial for addressing these complex and interconnected issues effectively [55].

The Antimicrobial Assessment on Global Aquaculture Production (AGAP), sponsored by the Monterey Bay Aquarium, represents a crucial initiative that convened a panel of experts to address the significant challenges associated with AMU in aquaculture. The discussions encompassed a range of vital themes, including (1) evaluating the current state of knowledge on AMU and identifying existing gaps; (2) formulating strategies to identify ecologically relevant impact indicators and establish thresholds for assessment; (3) identifying pivotal socioeconomic factors and effective governance mechanisms essential for implementing monitoring practices in aquaculture and extending them across sectors and countries to enhance the sustainability of aquaculture; (4) developing pathways to enhance our comprehension between ABU in aquaculture and AMR; and (5) exploring potential AB monitoring tools that can be universally adapted and implemented across region and sectors. By addressing these critical areas, the AGAP aims to foster informed decision-making and promote sustainable practices within the aquaculture industry on a global scale. The AGAP program designed a series of workshops to better understand how the role of ABU intersects with aquaculture production and serves as evidence of the power of collaboration in addressing complex global challenges to achieve sustainable aquaculture.

## 2. Results

### 2.1. Participants’ Subsection

**Figure 1:**
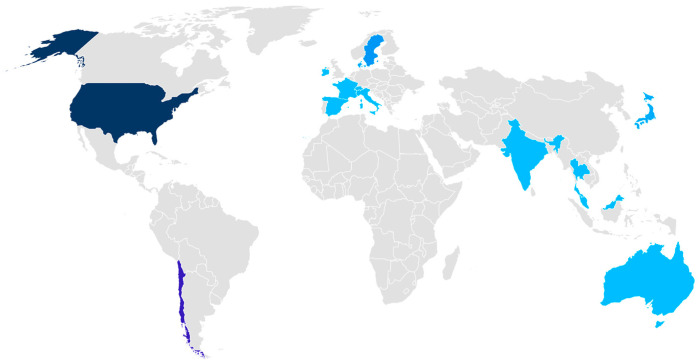
Global geographic representation of experts from global aquaculture representing countries (*n* = 20) across the globe.

Workshop attendees included 23.2% women and 76.8% men, representing five main sectors (academia—44.6%, non-governmental institutions (NGO)—19.6%, business—16.1%, international agency—14.3%, and governmental institutions—5.4%). There were representatives from 34 different affiliations from four continents (Europe, North and South America, Oceania, and Asia), and 20 countries, namely Australia (1), Chile (11), Denmark (2), France (1), Hong Kong (1), India (1), Ireland (1), Italy (1), Japan (2), Malaysia (1), Myanmar (1), Scotland (1), Spain (1), Sri Lanka (1), Sweden (3), Switzerland (1), Thailand (1), The Netherlands (3), the United Kingdom (4), and the United States of America (18) (Figure 1).

### 2.2. Workshop 1: General AMU and Ecological Impacts in Aquatic Ecosystem

**Figure 2:**
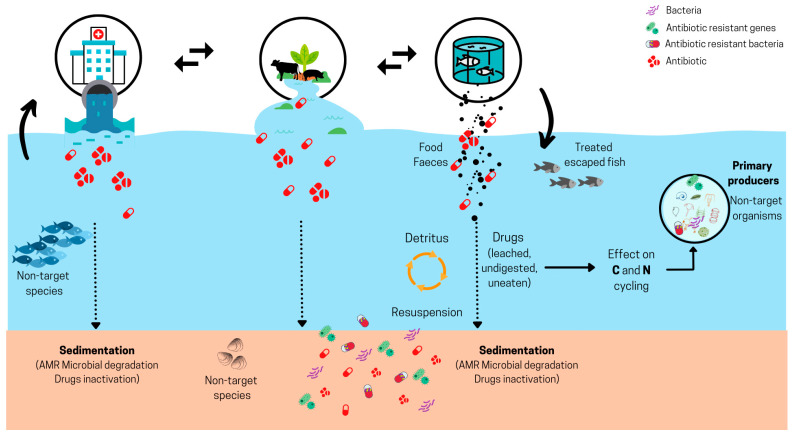
Discussed theoretical framework of possible antimicrobial (AM) dispersion in aquatic ecosystems. AMR: antimicrobial resistance, C: carbon, N: nitrogen. Adapted from [22,34].

During the discussion, a theoretical framework was introduced (Figure 2) to illustrate the potential ecological impact of AMU in aquaculture. The framework aimed to facilitate the assessments of possible ecotoxicological effects, determine risk, and review AMR. The experts indicate that the impact of AMs on the aquatic ecosystem is a complex problem, and there is limited information regarding their ecological effects. In this context, separating the impacts generated in freshwater and the marine environment is essential, given the different exposure routes, scenarios, and species potentially affected.

Effective surveillance is required to improve the current understanding of AMR, specifically in terms of monitoring the improvement toward proposed goals, identifying emerging issues, and understanding the harmful effects and toxicity that lead to microbial resistance. For instance, it was discussed that AMs mainly affect microorganisms, but the knowledge of how the disruption of microbial communities can affect the other organisms present in aquatic environments is also necessary. Because bacteria studies in the laboratory are different from environmental impact assessments in aquatic ecosystems, it is difficult to extrapolate the results to real environmental situations.

At present, theoretical assessment frameworks for farming animals (e.g., poultry) are valuable tools for measuring the potential effects of AMU. Nevertheless, different scales (e.g., spatial and temporal) and scenarios should be taken into consideration. Accordingly, conducting field studies is needed to obtain valuable data. Participants identified that the expected persistence and effects in the environment vary according to the chemical structure of AMs. Therefore, case studies in areas where AMs are highly used are needed to better understand the impacts.

As part of the discussion, it was revealed that, amongst non-target organisms, cyanobacteria and microalgae from freshwater can be affected by AMU, as proposed by Ve Van et al. [33]. The ecological risk of AMs can indeed be potentially higher in freshwater ecosystems compared to marine ecosystems, which is mainly caused by differences in the relative volumes of AM spread in the water and the access that humans have to freshwater, leading to greater contact probability, which contributes to this heightened risk. Overall, when unavailable, a prospective risk assessment framework for AMs should be incorporated into country policies. At present, these frameworks for aquaculture already exist in the United States, Japan, and the European Union, but they are in the early stages [1,56]. Nevertheless, more discussion and data records are needed, and the harmonization of risk assessment methods would be beneficial.

### 2.3. Workshop 2: Socioeconomic Perspective

AM usage (use, overuse, and misuse) in aquaculture extends beyond a technical problem. Additionally, socioeconomic drivers that comprise production include but are not limited to AM prescription practices, food safety concerns, marketing and consumer preferences, and the interaction with producers for effective use and governance.

The main findings for how behavior related to AMU can be changed and improved were based on a broader socioeconomic understanding. Experts discussed comparing aquaculture with land-based production such as poultry or pigs and indicated that aquaculture has a lower proportion of AM use, including [their use as] growth promoters. Attendees identified the importance of inaccuracies in AM and antibiotic use data since the reports are not coming from farmers.

The participants expressed concern regarding producers in developing countries, both small and large-scale producers, often not being trained (and informed) or regulated for AMU, which warrants attention. Producers often get easy access to products and sell products containing AMs that are not identified as such. As a result, they are not appropriately applied. Additionally, there are poor cost-effective alternative tools for managing diseases or subsidies for small-scale farms to implement good practices in aquatic animal health. This coincides with insufficient aquatic health professionals and veterinary services, as occurs in some Asian countries and Africa.

### 2.4. Workshop 3: Antibiotics in Aquaculture

The panel’s assessment underscores the complexity of identifying whether aquaculture serves as a source or a vehicle of AMR, for example, resistance generation or proliferation. This is mainly caused by the scarce existing evidence related to resistant bacteria in human clinical practice. Hence, this challenge is compounded by uncertainties regarding some inquiries that persist such as the real magnitude of AMR contributions from aquaculture activity in contrast to other sectors, such as land-based animal farming (e.g., poultry or pig farms), and human ABU.

Good sanitation practices and biosecurity at the farm level are indeed critical for reducing the need for ABs, minimizing bacterial infections, and mitigating the associated revenue losses in aquaculture operations. Sanitization conditions are prioritized to avoid the use of ABs and should be complemented with regulations and responsible and adequate use to control and reduce AB use in land-based animal farms. However, the environmental/water quality in aquaculture production is hard to control, especially in an open system.

In addition to good sanitation practices, certification schemes play a crucial role in ensuring product quality, safety, and integrity within the aquaculture industry. Presently, some of the certification schemes such as Best Aquaculture Practices (BAPs), Aquaculture Stewardship Council (ASC), and GLOBALG.A.P. could to some extent guarantee the sustainability and quality of the commodities regarding AMU. Yet, the percentage of certified sustainable products is significantly lower in aquaculture goods than in other sectors. There is little opportunity to differentiate products through pricing, and small-scale producers cannot access certification. For instance, in fisheries, organic-produced food is more expensive because large federal programs do not support it, while in land-based agriculture, corn, soy, and other cash crops are cheaper for the consumer.

Participants highlighted the differences among aquaculture producer countries regarding product handling practices, identifying that, in certain countries, farmers directly applied active AM compounds to feed and water with inappropriate dosages, meaning they have direct contact with the chemicals, while there have been no studies on the effect of chronic exposure to antibiotic handling. Such exposure could have relevant effects, for example, causing or enhancing allergies.

Experts agreed that there is no model for risk assessment to address the exposure to AMR bacteria in the different areas covered by the One Health approach (ecosystems, animals, and humans). However, Denmark is an interesting case study of how this could be pursued in aquaculture. The country has well-connected data with 3–4 antibiotics classes, susceptibility data, and pathogen types that are well documented, which is not the case for other AB consumer countries.

### 2.5. Workshop 4: Methods for Determining Impact

The dialogue among participants highlighted significant gaps in assessing AM impacts on ecosystems. One major challenge identified was, in general, the difficulty in these studies as there is a lack of a baseline for naturally occurring concentrations of AMs or frequencies of antibiotic-resistant genes (ARGs) in aquatic ecosystems. This absence of baseline data complicates efforts to quantify the extent of AM contamination and its potential ecological effects. Chemical persistence is dependent on many factors, including local abiotic factors. When AMs are discharged into the aquatic environment, they can be distributed over vast areas and travel from freshwater systems to coastal zones, where chemicals usually accumulate in sediment and persist for long periods.

Recommendations included the extrapolation of the models currently used for terrestrial animals to build a baseline. Yet, as no database is available to use models in aquaculture systems, it is, therefore, necessary to begin trials to obtain the essential data to run models (trials could be performed in the field or mesocosm).

During the conversations, experts indicated a recent in situ experimental assessment conducted by Gonzalez-Gaya et al. [57], in which the effects of aquaculture and AMs on marine ecosystems were evaluated based on an optimized method that assesses the risks of AMs, as well as their occurrence and accumulation. Their study showed the moderate persistence of flumequine and oxytetracycline in marine sediments and how uptake by macrofauna and benthic environment contamination and residual concentrations of AMs contribute to the selection of resistant genes. This methodology applied in the indicated investigation could be used as a basis to propose pilot studies.

### 2.6. Challenges and Recommendations

**Figure 3:**
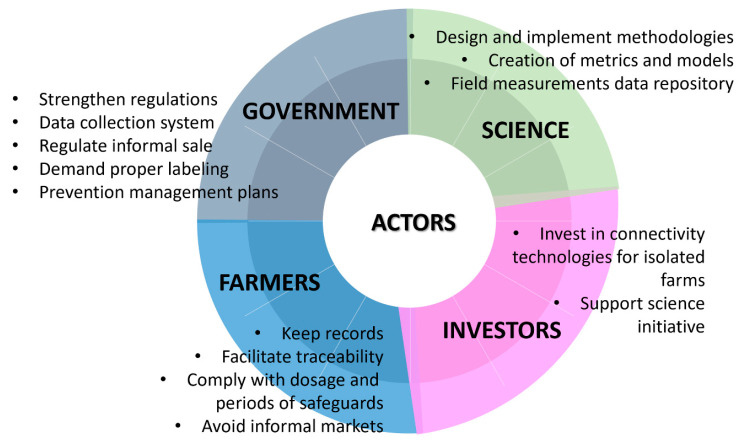
Identified gaps and their translation for stakeholders and extension agents, specifically four primary actors: government, farmers, investors, and scientists.

A series of knowledge gaps were identified in the AGAP series, as well as recommendations provided by experts (Table 1). Based on the results, it is recommended to transfer valuable information; thus, the identified gaps and recommendations from experts and their translation for stakeholders and extension agents, specifically with investors and governments as extension agents and farmers and scientists as stakeholders, play a relevant key role in the aquaculture value chain (Figure 3).

## 3. Discussion

Recognizing the implications of AMU in aquaculture implies finding measurable and suitable indicators and proper functional thresholds for measuring effects/impacts on aquatic ecosystems and AMR. Further information is also still needed for heightened awareness of AMR sources and their effects on the environment [28].

Caputo et al. [28] reviewed global research on the FAO National Actions Plans (NAPs) and found that some countries have committed to progress, yet gaps in the sources of AMR in aquatic organisms need further research. The recommendations advised by the panel of experts align with those described by the authors.

### 3.1. Antibiotics in Aquatic Ecosystems

Biogeochemical cycles such as nitrogen and carbon cycling regulated by bacteria might be affected by AMU, but limited information is available. Also, there is insufficient information about ecological or environmental impacts on non-target aquatic organisms (e.g., benthic fauna). Consequently, pollution evaluations are required, which should include evidence regarding monitoring strategies for water and sediments. Ideally, these evaluations should also encompass non-target organisms such as shellfish, fish, and wild species. From the experts’ outlook, marine and freshwater must be treated individually as they are different systems with distinct environmental conditions.

Accurate information concerning AMU use patterns, as well as quantities and emissions of AMs, is indispensable for effective management and for measuring the impacts of AMs. Regarding regulatory expertise, there is a noticeable absence of evidence concerning ecotoxicological impacts. While there currently are environmental quality standards (EQSs) for the environment and emissions that could serve as valuable guidance in aquaculture, the current environmental risk assessment (ERA) framework for drug authorization is not adequate for assessing environmental risk. Moreover, there is also a concomitant reduction in information transfer from developed to developing countries. Consequently, it is imperative to develop and include ecological evaluation criteria for AMs in national policies.

In discussing socioeconomic behaviors and their underlying drivers, the workshops acknowledged the complexity of the topics and emphasized the need for evidence to conduct investigations and build a knowledge base to further identify the crucial drivers and strategies to control them. The primary findings highlight the pressing need for more precise and robust regulatory frameworks and training resources to guide farmers, especially small-scale farmers, as these groups of producers often face challenges in accessing training and understanding of disease risk and certification management schemes. Some of these issues, for instance, reliable ABU data, access to professionals, and poor regulation, have been identified in shrimp farms [13].

Farmers who operate under a lower level of technical knowledge have poorer practices for AMU. For instance, shrimp farmers are challenged by poor access to treatments, specialist advice, or facilities to treat sick shrimp [13]. The need for professionally trained veterinarians is crucial, as professionals are able to guide good practices, implement protocols, contribute knowledge, and provide support to farmers [11,58]. Similar findings were identified by Lambraki et al. [59], who recommended providing training for the workforce across sectors.

Improving the biosecurity system in farms will promote and ensure animal health. Thus, biosecurity has been considered a valuable tool for animal welfare, and together with the collaboration between veterinarians, farmers, and researchers, it will be possible to strengthen best management practices [58,59], which are not limited to aquaculture production.

Extension services have previously been strongly recommended for shrimp aquaculture [27,38] and suggested to be part of the existing surveillance of diseases in aquatic systems [16]. They play a critical role in strengthening appropriate practices regarding AMU, implementing regulations, translating science knowledge for farmers, and developing and applying suitable monitoring tools for risk assessment [56,60]. Clear communication between producers and the market segmentation is needed, given the different scales, objectives, and risks associated with each segment, and along with these, the potential impact of different actions.

A view based on trust would foster effective communication between producers and regulators, facilitating open and transparent communication channels for sharing information for which extension services could be supportive. This will reinforce the support acknowledged by small-scale producers, resulting in a clearer understanding of AMs as a productive tool and better disease management practices, thus reducing their needless use and contributing to the sustainable development of the aquaculture sector.

Methodological development is not the only area that will benefit from further research. While there is a general understanding of what drives are behind AMU and misuse in aquaculture, there are critical data gaps in AM usage (i.e., quantification of amount and type of AMs used) across sectors and how they are distributed among major producer countries. The lack of robust data collection, analysis, metrics, and governmental initiatives to obtain this information underscores the urgent need for scientific studies to assess the impacts of AMU on the ecosystem [11]; however, a lack of funding hinders efforts in conducting scientific studies. However, the necessity of scientific evidence in alliance with policymaking has previously been suggested [61], and studies are still needed to bridge the gap. Standardized methods have been described as critical to ensuring comparative results between different studies [13,62], there are inappropriate methods for testing AM susceptibility [63,64], and data sharing and collaboration are essential within industries and governments to bridge positive actions in different sectors to confront AMU/AMR [59] and work through interdisciplinary research toward responsible and sustainable AMU.

### 3.2. General Recommendations and Actions Taken

The recommendations identified in the AGAP meetings and their translation among stakeholders are very important, as they are not usually part of the discussions. It is necessary to strengthen efforts to incorporate them into the circle. These actions will ideally initiate efforts to tackle AM issues [59]. In a recent review, Ibrahim et al. [65] indicated similar trends as there are still insufficient statistics on AMU in global aquaculture. Land-based farmed animals from European countries have competent data available, mainly attributed to well-established surveillance, monitoring systems, and networks [58].

Risk assessment frameworks mandate a critical understanding of the connectivity between the aquaculture systems and the people living in the area. Such frameworks will allow for the development of evidence-based future action plans, including fieldwork on selected aquaculture-relevant countries, and include suggestions to non-profit institutions, civil society, governments, and the aquaculture industry. The alignment between different actors is critically required, starting from the basic research, translation, and application [11,59,61].

Some examples of actions taken by different initiatives are as follows: For instance, Chile has three initiatives for investigating AB reduction with solid data, namely the CSARP program (private, www.csarp.cl), the Pincoy project (private, www.proyectopincoy.com), and PROA (governmental, www.sernapesca.cl). This country is a good example of initiatives with well-established and reliable data on ABU in the salmon industry. Other initiatives, such as SeaBos (European Union, www.seabos.org), collaborate across sectors within the global seafood industry, one of the tasks of which is AB reduction. Another initiative was implemented in 2022 by the WOAH, which released the Animal Antimicrobial Use (ANIMUSE) (http://amu.woah.org), a platform that aims to facilitate access to AMU information worldwide, but only 11 out of 183 countries report specific quantities of AB consumption, with no species indication, and 60 countries report aquaculture use combined with terrestrial use. Other initiatives focused on AMR include ReAct (www.reactgroup.org), an independent international network, and ICARS (www.icars-global.org) from Denmark, which enables investigating AMR mitigation. Also, some certification programs such as the Aquaculture Stewardship Council (ASC) and Best Aquaculture Practices (BAPs) have mandatory records. However, the main problem is related to small-scale aquaculture where no incentives and certification exist, and where veterinarian services are not provided. Besides some initiatives taken in some countries to improve AB management, data tracking, and antibiotic reduction programs, accurate information for improving knowledge on ABU in aquaculture is still missing. This event succeeded in providing an avenue for effective networking among worldwide experts to work together on future research and improve knowledge of ABU in aquaculture.

### 3.3. Participatory Experts’ Initiative

International online experts’ meetings are a great opportunity to share knowledge and strengthen connections. Weitzman et al. [66] presented valuable information regarding carrying capacity in aquaculture based on holistic assessment. Lambraki et al. [57] demonstrated the value of participatory workshops for discussing AMR in European food systems, and Salgado-Caxito et al. [67] showed an interesting holistic qualitative risk assessment method for AMR within the production of salmon. For our part, the effort to address key aspects of AMU in aquatic ecosystems allows us to contribute to providing valuable information regarding knowledge gaps and methodology assessment by establishing ecological indicators and thresholds, effective antimicrobial governance, socioeconomic considerations, and monitoring approaches in the context of aquaculture.

The international workshop took place during the global COVID-19 pandemic, and despite the challenge, AGAP demonstrated the success of online communication and effective knowledge exchange, fostering collaboration and advancing understanding on this critical topic, and marked an important step in the right direction to tackle the impact of ABs used in aquaculture. Nevertheless, it is important to acknowledge the limitations of the event. Although diligent efforts were made to connect with global experts from various disciplines, constraints such as time limitations, geographical barriers, online connectivity issues, and even language considerations may have inadvertently narrowed the possibility of having more experts attending the event. Other relevant topics may have been left unaddressed, but they are needed, such as the necessity of investment in prevention as vaccines’ development will make a difference in reducing the impact that ABU can have, or the necessity of funding for ecological studies.

## 4. Materials and Methods

Initially, the strategy consisted of a systematic literature review, which was conducted to evaluate the preliminary information on the topic and the existing knowledge gaps. The review covered an extensive range of research, including peer-reviewed publications (*n* = 112), reports (*n* = 9), and relevant websites (*n* = 4), which were examined to evaluate the preliminary information available on the topic, the main unsolved issues, and the existing knowledge gaps. References were retrieved from the Web of Science, covering papers published between 1987 and 2022. The keyword combinations in the search included “aquaculture AND (antibiotic OR antimicrobial) AND (resistance OR consumption OR dose OR dosage OR use OR residues OR reservoir) AND (shrimp OR salmon OR prawn OR tilapia OR catfish)”. This led to the identification of the four main topics to discuss during each workshop, namely (1) general AMU knowledge and ecological importance, (2) the socioeconomic perspective, (3) ABs and ABU in aquaculture, and (4) methods for determining impacts.

**Figure 4:**
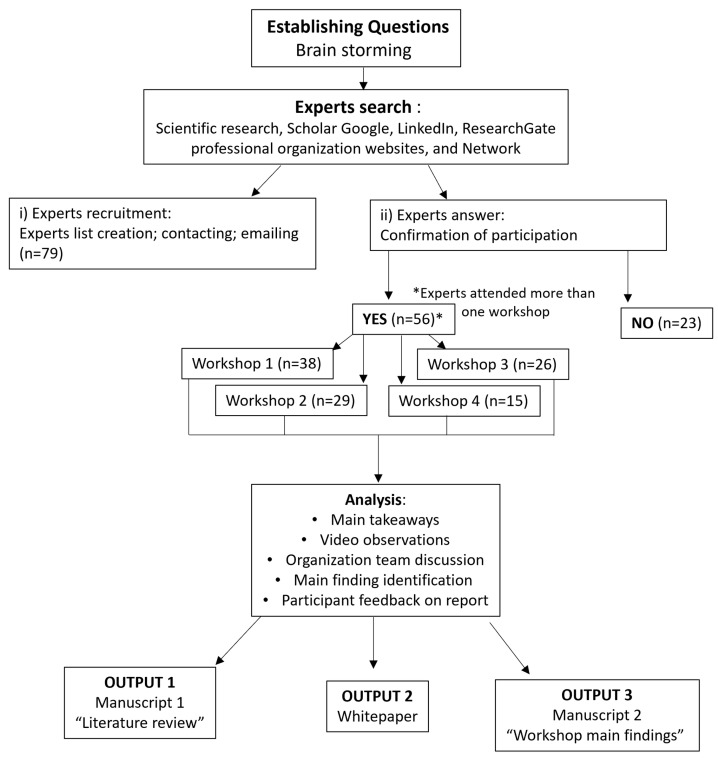
Diagram of the methodology applied for workshops’ organization, procedure, and output design.

All participants’ consent was sought via email prior to their involvement, and during the workshop event, they were explicitly informed (verbally) of the recording process and prompted to consent by clicking the “accept” button. The participation was voluntary, and attendees were assured of their autonomy to withdraw from the study at any given point. The information provided by the experts was related to their academic or technical expertise. Additionally, a final report was disseminated to all participants for their review and feedback (Figure 4).

The inclusion of experts with diverse perspectives and backgrounds ensured a comprehensive and robust exchange of ideas and insights during the thematic discussion panels. The organizer team was established with the integration of equality, diversity, and inclusion principles to avoid any bias and implications on the results. The study refrained from any involvement in the use of personal data and/or sensitive content, data of private origin, and collection and/or analysis of human data.

### 4.1. Participants

The process of identifying participants for the thematic discussion panels involved a comprehensive approach utilizing various channels and methodologies (Figure 4). After the four main topics were identified, and pertinent questions were formulated, experts from diverse areas of knowledge were identified through extensive search across scientific research and professional organization websites, as well as academic and non-academic platforms such as LinkedIn, Google Scholar, and ResearchGate, using a general snowballing methodology. Subsequently, potential participants were selected according to their area of expertise, aquaculture AM background, involvement in governance decision-making agencies, networking colleagues, and business partners. A total of 79 global aquaculture experts (finfish and shrimp/prawn aquaculture) were initially approached via email, inviting them to participate in the thematic discussion panel. Out of the 79 experts, 23 either rejected the invitation or did not respond. Consequently, a diverse group of 56 worldwide experts from 20 countries participated in the four thematic discussion panels from diverse perspectives such as ecology, microbiology, economics, aquaculture, AMR, and other environmental sciences. Some experts attended more than one workshop; thus, between 15 and 38 experts attended each workshop.

### 4.2. The Workshops

The online meetings (3 h in length) were carried out throughout 2021 and early 2022. Participants were asked to be recorded, and all of them provided their consent and were informed about the outputs after the workshops. The modality of the workshops was an open discussion format, with initial questions provided to start the discussion and encourage participants to share their opinions, identify central issues, propose suggestions, and recommend guidelines for the designated topic (Table 2). Each workshop started with a welcoming section and agenda presentation, followed by a group photo; the online events were led by a facilitator who guided the discussions and ensured that all participants had the opportunity to contribute their perspectives and insights.

After each workshop, the main takeaways were taken. When the AGAP event was finalized, the video records were examined, information was transcribed, and the information was discussed with the organizing team to generate outputs and the final report. The information was sent to experts to provide feedback (Figure 4).

## 5. Conclusions

Global experts contributed invaluable knowledge within their field, providing comprehensive insights across the diverse areas covered. Aligned with the proposed objectives, it was possible to obtain valuable information and a roadmap to establish investigation priorities, improve standards, foster integrated thinking approaches, strengthen knowledge, and build capacity on the relevant topic of ABU within the aquaculture system. Despite the diligent efforts made to connect with global aquaculture experts from various disciplines, some constraints narrowed the possibility of covering relevant topics that are needed, such as the necessity of investment in preventive measures such as vaccines, capacity building, appropriate antimicrobial susceptibility testing methods, or resistance genes that can make a difference on reducing the impact of ABU.

Important challenges remain in the research and policy areas to improve the use and reduce the impact of ABU in aquaculture and move toward a more sustainable industry. Overall, the workshop evidenced the power of collaboration in addressing the complex global challenges of antibiotic use in aquaculture.

## Figures and Tables

**Table 1:** Critical gaps identified in the Antimicrobial Assessment on Global Aquaculture Production (AGAP) series of workshops. Abbreviations include AB: antibiotic, ABU: antibiotic use, AM: antimicrobial, AMR: antimicrobial resistance, ARG: antimicrobial-resistant gene, ERA: environmental risk assessment, EQS: environmental quality standards.

DIMENSION	DRIVER, GAPS	GENERAL RECOMMENDATIONS
ANTIBIOTIC USE: methodological and technical for assessing ecological impacts		
Measuring impacts	Indirect impacts on the environment must be assessed for conducting environmental evaluations (e.g., ecological processes intervened by bacteria present in the environment or benthic organisms affected by ABs).	In the absence of clear information on the ecological effects caused by AMs, it is prudent to differentiate freshwater and marine environment impact assessments. Ecological assessment criteria for ABs should be incorporated into national policies.
	Absence of models on cumulative impact (e.g., multiple sources or chronic discharge); validated models are required for evaluating impacts and delivering recommendations.	Implementing surveillance tools (e.g., benthic respiration, oxidative stress, oxygenation, primary production, environmental DNA, metabarcoding, metagenomics, and others) is required.
	Impact thresholds should be established.	The existing ERA used for drug approval is not enough for assessing environmental risks; therefore, implementing or improving ERA is necessary.
Methodology	Lack of standardized methodology for measuring in situ ecological impacts; the methodology that is currently used is expensive and not standardized.	Conducting pilot assessment/field tests in key water bodies (e.g., fjords) is needed for obtaining valuable real data and determining impacts, as it is vital to have knowledge on measuring ecological impacts since an evaluation framework does already exist.
Monitoring	Missing information on AM passive monitoring in aquatic environments.	EQS (AMR and ecotoxicological) for emissions and the environment can serve as a valuable reference point in aquaculture.
**ANTIBIOTIC USE AND ECOLOGICAL IMPACTS: regulatory aspects**		
Environmental Quality standards	Absence of international agreement and validation of EQS (i.e., AMR and ecotoxicological) for AM release.	
	The current ERA used for drug approval is insufficient to assess environmental risk.	
Risk assessment	Deficiency in standard risk assessment methodologies appropriate for developing countries, and barely applicable for aquaculture; risk assessment methodologies for pathogen risk analyses are well developed in aquatic food production systems.	
Protection	Specific safety objectives for human health and ecosystems should be outlined.	
**SOCIAL AND ECONOMIC**		
Economic incentives	Low-cost access in informal markets.	Identify informal sources of access to antibiotics and access to certifications for the proper use of antibiotics and design subsidies for small farmers that can demonstrate traceability in ABU.
Regulations	Inappropriate regulations to control the overuse of ABs and informal access.	Strict regulations should be established in each country regarding the sale, use, and monitoring of ABU in aquaculture. There should be more surveillance on the application of the regulation as there exist international documents that some countries do not apply.
Farmers’ attitudes and knowledge	Lack of risk perception and awareness of impacts of AB misuse.	Public communication programs for behavioral change. Research in stakeholders’ preferences and behaviors.
	Lack of training in the sustainable use of ABs and disease management.	Improved small-scale disease management tools. Characterization of producers and identification of their knowledge gaps on the use of ABs and their impacts. Improve coordination between farms in the same zone.
Biosecurity	Restricted access to freshwater and poor sanitation.	Training for farmers and field personnel is essential to improve biosecurity at a farm level. More trained personnel, ideally Veterinarians, to guide practices on AM and AB use.
	Lack of professional veterinary services.	
	Scarcity of laboratory infrastructure in microbiological testing.	
	Inadequate access to AB.	Improve the technique of AB selection according to the type of disease.
	Lack of reporting on ABU and monitoring.	Need for traceability and tracking systems for the use of AB.
**THE ROLE OF AMR ON AQUACULTURE**		
Scientific research	While recent scientific research has emerged, substantial gaps in understanding AMR persist.	To collect more knowledge to answer relevant questions regarding AMR. More investigation is needed and the funding to conduct investigations.
	Better knowledge is required regarding the relative magnitude of aquaculture contrasted with land-based animal farming systems/humans’ AM (over-)consumption.	
	Absence of detailed information about naturally occurring ARG to develop methodologies to establish impact.	
Aquaculture systems boundaries	Deficient knowledge of how to determine aquaculture systems boundaries.	Strengthening knowledge of the role of AMR is fundamental.
Hazard	There is a need to categorize systems regarding hazards.	Enhancing knowledge of the theme.

**Table 2:** Workshop series organizational scheme. AB: antibiotics, ABU: antibiotic use, AGAP: Antimicrobial Assessment on Global Aquaculture Production, AM: antimicrobial, AMU: antimicrobial use, AMR: antimicrobial resistance, ARG: antimicrobial-resistant gene, FAO: Food and Agriculture Organization, WOAH: World Organization for Animal Health.

	Workshop 1 General AMU and Ecological Impacts	Workshop 2 Socioeconomics Perspective	Workshop 3 ABs in Aquaculture	Workshop 4 Methods for Determining Impacts
**Date**	27 May 2021	22 July 2021	23 September 2021	2 February 2022
**Objective**	Evaluating the current state of knowledge on AMU and identifying existing gaps. Formulating strategies to identify ecologically relevant impact indicators and establish thresholds for assessment.	Identifying pivotal socioeconomic factors and effective governance mechanisms essential for implementing monitoring practices in aquaculture and extending them across sectors, and countries to enhance the sustainability of aquaculture.	Developing pathways to enhance our understanding of AB usage in aquaculture and AMR.	Exploring potential AB monitoring tools that can be universally adapted and implemented across regions and sectors.
**Attendees**	38 attendees among experts (26), guests (4), and the AGAP team (8) from 22 institutions worldwide belonging to 11 countries; 71.1% men and 15.5% women	29 attendees among experts (20), guests (5), and the AGAP team (4). The attendees were representatives of 10 countries and 15 institutions, including the FAO, WorldFish, and WOAH. 75.9% men and 9.2% women.	26 attendees, among experts (17), the organizing team (3), and guests (6) from 14 institutions, including FAO, WorldFish, and WOAH, representing 13 countries, with 69.2% men and 11.6% women.	15 attendees among the AGAP team (5) and experts (10) from 9 institutions belonging to 4 countries, with 60% mean and 10% women.
**Field**	Land-based animal growing systems, freshwater aquaculture, and marine-based aquaculture, as well as fields involving pathologists, animal health experts, ecologists, food services, and ecotoxicologists, were among other areas of expertise concerning prawn farms and fish farms.	Animal health experts, ecologists, experts in certification and food systems, livestock specialists, socioeconomists	Pathologists, epidemiologists, microbiologists, and data management and aquaculture system experts	Experts involved in pharmacology, food safety, and animal health, as well as environmental experts, researchers using analytic techniques, and ecotoxicologists
**Duration**	3:30 h	3:00 h	3:00 h	3:00 h

## Data Availability

All relevant data are within the manuscript.
